# The Association between the Extent of the Osteoarthritic Meniscus Degeneration and Cigarette Smoking—A Pilot Study

**DOI:** 10.3390/medicina60020323

**Published:** 2024-02-14

**Authors:** Maria Zabrzyńska, Maciej Pasiński, Maciej Gagat, Michał Kułakowski, Łukasz Woźniak, Karol Elster, Paulina Antosik, Jan Zabrzyński

**Affiliations:** 1 Faculty of Medicine, Collegium Medicum in Bydgoszcz, Nicolaus Copernicus University in Toruń, 85-067 Bydgoszcz, Poland; 2 Department of Orthopaedics and Traumatology, Faculty of Medicine, Collegium Medicum in Bydgoszcz, Nicolaus Copernicus University in Toruń, 85-067 Bydgoszcz, Poland; pamacos2014@gmail.com (M.P.); zabrzynski@gmail.com (J.Z.); 3 Department of Histology and Embryology, Faculty of Medicine, Collegium Medicum in Bydgoszcz, Nicolaus Copernicus University in Toruń, 85-067 Bydgoszcz, Poland; mgagat@cm.umk.pl; 4 Faculty of Medicine, Collegium Medicum, Mazovian Academy in Płock, 09-402 Płock, Poland; 5 Independent Public Healthcare Center in Rypin, 87-500 Rypin, Poland; mkulakowski@poczta.fm (M.K.); karol.elster@gmail.com (K.E.); 6 Department of Orthopaedics and Traumatology, University of Medical Sciences, 61-701 Poznan, Poland; 7 Department of Clinical Pathology, Faculty of Medicine, Collegium Medicum in Bydgoszcz, Nicolaus Copernicus University in Toruń, 85-067 Bydgoszcz, Poland; paulina.antosik@cm.umk.pl

**Keywords:** meniscus, meniscus tear, osteoarthritis, smoking, Bonar score system, varus knees

## Abstract

*Background and Objectives*: The negative effects of smoking on the musculoskeletal system were presented by many authors, although the relationship between smoking and osteoarthritis remains unclear. The aim of this paper was to investigate the negative effects of smoking on meniscal tissue in osteoarthritic knees by microscopic examination, by adapting the Bonar scoring system and its modifications. *Materials and Methods*: The study involved 34 patients with varus knees, from whom 65 samples of knee menisci were obtained. The mean age in the studied group was 65.385 years. The smoking status of the patients concluded that there were 13 smokers and 21 nonsmokers. *Results*: Among smokers, the mean classical Bonar score was 8.42 and the mean modified Bonar score was 6.65, while nonsmokers were characterized by scores of 8.51 and 7.35, respectively. There was a statistically significant negative correlation between the number of cigarettes and the collagen in the medial meniscus (*p* = 0.0197). Moreover, in the medial meniscus, the modified Bonar score correlated negatively with the number of cigarettes (*p* = 0.0180). Similarly, such a correlation was observed between the number of cigarettes and the modified Bonar score in the lateral meniscus (*p* = 0.04571). Furthermore, no correlation was identified between the number of cigarettes and the classical Bonar score in the lateral meniscus. There was a statistically significant difference in the collagen variable value between the smokers and nonsmokers groups (*p* = 0.04525). *Conclusions*: The microscopic investigation showed no differences in the menisci of smokers and nonsmokers, except for the collagen, which was more organized in smokers. Moreover, the modified Bonar score was correlated negatively with the number of cigarettes, which supports the role of neovascularization in meniscus pathology under the influence of tobacco smoking.

## 1. Introduction

**Figure 1:**
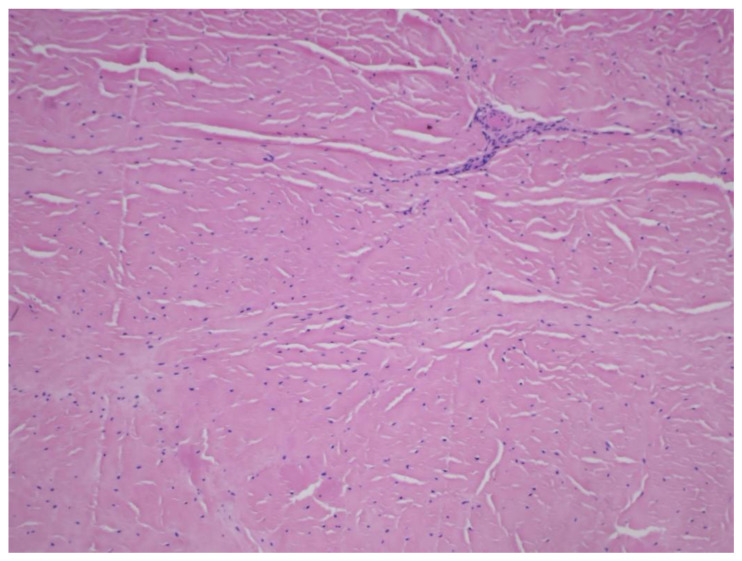
Microscopic evaluation of the meniscal sample stained with H&E showed a red–white zone of the menisci.

Nowadays, there is a growing interest and understanding of the knee menisci and their crucial role in maintaining proper knee joint congruence and kinematics, despite the fact that in the past they were considered to be functionless, vestigial structures and were often excised with an open total meniscectomy [1,2,3]. The menisci can be described as a fibrocartilaginous, crescent-shaped wedge structure located between the condyles of the femur and tibia [2,4]. They cover 75% to 93% of the articular surface of the corresponding tibial plateau, forming the acetabulum of the knee joint [1,5]. Their function allows them to maintain proper knee joint performance and biomechanical functions. They are mainly involved in load-bearing, load transmission, and shock absorption. Moreover, they participate in proprioception, articular cartilage nutrition, lubrication, and protection. They are also important passive joint stabilizers (Figure 1) [2,6,7].

The Bonar score system is the most common and established scale to assess the microscopic evaluation of tendon pathology. This semiquantitative scoring system has been modified many times in order to improve diagnostic and microscopic evaluation [8,9,10,11,12,13,14]. Thus, it can be modified to quantify the pathological changes in meniscal tissue. Park et al. were the first to present the use of the Bonar score in meniscus root pathology in knee osteoarthritis (OA). Their study presented the similarity between meniscus root fibrous connective tissue, and tendons connective tissue microstructure [15].

Osteoarthritis is characterized by knee cartilage volume loss and structural modification of cartilage, subchondral bone, Hoffa’s fat pad, synovial membrane, ligaments, and muscles. All of these features distinguish OA as a whole-joint disease [16,17]. Meniscal damage or meniscal excision are known as primary risk factors for rapid cartilage loss [18,19]. Both the excision of the meniscus and meniscal tears are factors resulting in cartilage lesions leading to knee joint OA [20]. Meniscal tears can easily lead to OA, and, on the other hand, osteoarthritis knees may develop meniscal tears. However, healthy menisci rarely occur in knees with OA [21]. Early knee OA is associated with either meniscal tears or symptoms such as synovial inflammation. Synovial inflammation is known as a major risk factor for OA’s rapid progression. Meniscal tissue with synovial inflammation differs with an enhanced level of metalloproteinase-13, which plays a major role in the degeneration of cartilage tissue in OA [22]. Menisci in OA knees display signs of pathological changes. They are often torn, macerated with cracks, have structural disorganization and fractures, or are even completely damaged, rather than without signs of degeneration [21]. Awareness of their importance prompts people to avoid meniscectomy and encourages the evolution of modern treatment methods for meniscal repair in order to save meniscal tissue [23,24].

Even though meniscal injuries are the second most common trauma to the knee, the data are still lacking in epidemiology and prognostic factors [23]. Blackwell et al. observed that current smokers were significantly more likely to undergo meniscectomy shortly after meniscus repair [25]. The negative association of smoking and pack-years with knee joint cartilage suggests that cartilage loss and defect may be inducted by smoking [26,27,28,29]. On the other hand, a few authors showed no negative association between smoking habits and meniscal tissue [30,31,32,33]. Nevertheless, smoking is commonly known as an important negative factor in orthopedics. Tobacco smoking results in decreased healing, complications, and poor postoperative outcomes in orthopedic surgery [23,34]. The relationship between smoking and OA remains unclear. Tobacco users may have an enhanced risk of OA due to cartilage damage and the ongoing inflammation process [35]. Nicotine, one of the constituents of tobacco smoke, promotes chondrocyte proliferation, migration, capillary formation, and decreased collagen synthesis [36,37]. Moreover, it stimulates the neovascularization process, which is known to take part in OA [38]. However, the effects of smoking on meniscal tissue have not been well explored yet. However, many authors present the negative effects of smoking on the musculoskeletal system, although there are still papers that undermine these outcomes. We hypothesized that the known negative effects of nicotine, such as neovascularization and stimulating inflammation, may negatively affect meniscal tissue healing. It would lead to the degeneration of meniscal tissue, which could be quantified using the Bonar scoring system. Thus, the aim of this study was to assess degeneration of the meniscal tissue in OA knees in correlation with smoking using the Bonar score in microscopic examination. Smoking may turn out to be an important negative factor in the degeneration of meniscal tissue in the OA knees. In this study, we attempted to investigate the negative association between smoking and meniscal tissue.

## 2. Materials and Methods

The study followed the principles outlined in the Declaration of Helsinki for experiments involving human subjects. Before the commencement of the study, approval was obtained from the local institutional Bioethics Committee (approval number KB 131/2022). The study included consecutive patients who underwent total knee arthroplasty for symptomatic OA between 2022 and 2023. All participants included in the survey were preoperatively diagnosed with gonarthrosis based on a clinical examination, as well as imaging modalities such as X-rays or magnetic resonance.

Inclusion criteria included the presence of severe unilateral OA with varus knee deformity (Kellgren–Lawrence score II or more), rheumatological diseases, and informed consent from the patient. Genu varum malalignment is characterized by <4° valgus knee axis or mechanical axis falling medial to the center of the knee. The following exclusion criteria were selected: secondary OA, previous surgical procedures within the affected knee, valgus deformity of the knee joint, severe deformity (>20 degrees varus), advanced OA in other joints, diabetes, advanced atherosclerosis of the lower limbs, cancer, and immunological diseases.

Patient demographic data, preoperative ROM, and preoperative X-rays (a long leg standing X-ray and an AP, lateral X-ray of both knee joints) were recorded. Written informed consent was obtained from all patients before they participated in the study.

The patient population was categorized as smokers or nonsmokers (those who had never used any nicotine supplement, such as nicotine gum or patch, oral snuff or moist snuff, cigars, or cigarettes). Also, dose-dependent and time-dependent data about smoking habits were collected, including the period of cigarette smoking (smoking years), the mean number of cigarettes smoked per day, and the pack-years index (one pack contains 20 cigarettes in Poland. The surgeons were blinded to the smoking status of the patients.

### 2.1. Surgical Technique

Total knee arthroplasty was performed in each case. All procedures were performed using an anteromedial approach; a tourniquet was applied in each case. In patients, the infrapatellar fat pad was bluntly divided from the patellar ligament and resected using electrocautery. However, surgeons incised it at the medial border of the patellar ligament to gain exposure to the knee joint. In all cases, the anteromedial joint capsule was routinely released from the tibia. The surgeries were performed following the concept of mechanical alignment; femoral components were implanted using the posterior referencing technique, while the rotation of tibial components was established parallel to a line drawn from the posterior cruciate ligament to the medial third of the tibial tuberosity. In all cases, the patella was neither resurfaced nor denervated, although large patellar osteophytes were removed if present. During the surgery, the menisci (both lateral and medial in each case) were dissected totally to preserve their original shape.

### 2.2. Histopathological Assessment

The menisci samples were fixed in 10% buffered formalin that was fresh and sterile. The samples were prepared using the hematoxylin and eosin (H&E) staining method, as well as the Alcian blue protocol. They were examined under light microscopy (Olympus BX46, Tokyo, Japan) using 5 μm sections. Alcian blue staining was explicitly employed to inspect the presence of ground substance glycosaminoglycans. It was carried out in accordance with the alcian blue protocol, respectively: deparaffinization of slides and hydrating to distilled water, staining in alcian blue solution for 30 min, washing in running tap water for 2 min, rinsing in distilled water, counterstaining in nuclear fast red solution for 5 min, washing in running tap water for 1 min, dehydrating through 95% alcohol, 2 times changing of absolute alcohol for 3 min each, clearing in xylene, and, in the end, mounting with resinous mounting medium.

The microscopic evaluation was carried out by two experienced observers who specialized in connective tissue. They were blinded to the identity of the samples. The extent of histopathological changes was assessed based on the classical Bonar score assumptions and their modifications. The classical Bonar scoring system evaluates four main variables: fibroblast/chondrocyte morphology, accumulation of ground substance elements, neovascularity, and collagen architecture. A scoring range of 0 to 3 points was assigned to each variable, with 0 indicating normal tissue and 3 representing extreme pathology. An utterly normal tissue would score 0, while a severely degenerated tendon would score 12.

In the second step of the examination, meniscal samples were evaluated using the modified Bonar scores developed by Zabrzyński et al. [14]. In this modified scoring system, a new Bonar score, the attributes of the neovascularization variable in the original Bonar scale were reversed. A score of three points was assigned to normal tissue with minimal occurrence of blood vessels (absent neovascularization), two points for the incidental presence of capillary clusters of less than one per 10 high-power fields (HPFs; mild neovascularization), one point for 1–2 clusters per 10 HPFs (moderate neovascularization), and zero points for more than two clusters per 10 HPFs (abundant neovascularization).

### 2.3. Statistical Analysis

Group comparisons and statistical analyses were conducted by two independent investigators using GraphPad Prism version 8.0.1 for Windows, GraphPad Software, Dotmatics, UK, www.graphpad.com (accessed on 24 December 2023). A *p*-value less than 0.05 was considered statistically significant. The normality of the variables was assessed using the Shapiro–Wilk test. Relationships between the studied parameters were evaluated using Spearman’s rank correlation coefficient. According to the nonnormal distribution of the data, intergroup comparisons were performed using nonparametric tests, specifically the Mann–Whitney U-test, for comparing two groups.

#### Correlation Analyses

The results of the Bonar and new Bonar scores were correlated with smoking indexes such as smoking years, the mean number of cigarettes smoked per day, and the pack-years index.

## 3. Results

The study involved 34 patients with varus knees, from whom 65 samples of knee menisci were obtained. The mean age in the studied group was 65.385 years (range: 54–81; SD = 6.88) at enrollment; the gender distribution was 21 women to 13 men. The demographic data are presented in Table 1.

By smoking status, there were 13 smokers (including 14 women and 7 men) and 21 nonsmokers (including 7 women and 6 men). No patients changed their smoking habits during the follow-up. Specifically, the mean age in the smoker group was 64.231 (range 54–72, SD = 6.0286) and 66.154 (range 55–81, SD = 7.3683) in the nonsmokers group (Table 1).

The mean classical Bonar score was 8.4462 (range 4–12, SD=1.5715). The mean modified Bonar score, which includes the reversed neovascularization variable, was 7.0462 (range 3–11, SD = 1.7804). Among smokers, the mean classical Bonar score was 8.4231 (range 6–12, SD = 1.6775) and the mean modified Bonar score was 6.6538 (range 3–10, SD = 1.8318), while among nonsmokers it was 8.5128 (range 4–11, SD = 1.5706) and 7.3590 (range 4–11, SD = 1.7089), respectively (Table 1).

The mean classical Bonar score for the medial menisci was 8.5000 (range 4–11, SD=1.5240), and for the lateral menisci, it was 8.3939 (range 6–12, SD = 1.6382). Regarding the modified Bonar score, the mean score for the medial menisci was 6.9375 (range 3–10, SD = 1.7586), and for the lateral menisci, it was 7.1515 (range 3–11, SD = 1.8221) (Table 2).

The mean number of cigarettes smoked per day was 5.2308 (range 0–25); for the medial menisci group it was 7.5 (range 0–25, SD = 9.5883), and for the lateral menisci group it was 3.0303 (range 0–25, SD = 6.9529) (Table 2).

**Figure 2:**
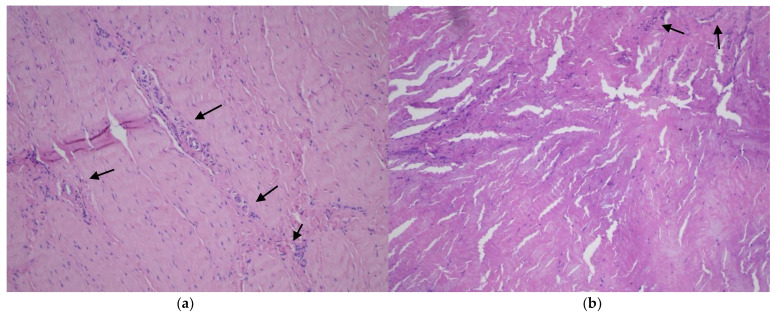
Microscopic evaluation of the meniscal sample stained with H&E. (**a**) Red–white zone in smokers groups with randomly scattered clusters of vessels (arrows). (**b**) Red–white zone in nonsmokers group with randomly scattered clusters of vessels (arrows).

Macroscopically, all examined menisci displayed signs of degeneration, including cracks, structural disorganization, and fractures. Histological examination of the meniscal specimens under a light microscope revealed tissue degeneration in all cases, both in the medial and lateral menisci (Figure 2).

**Figure 3:**
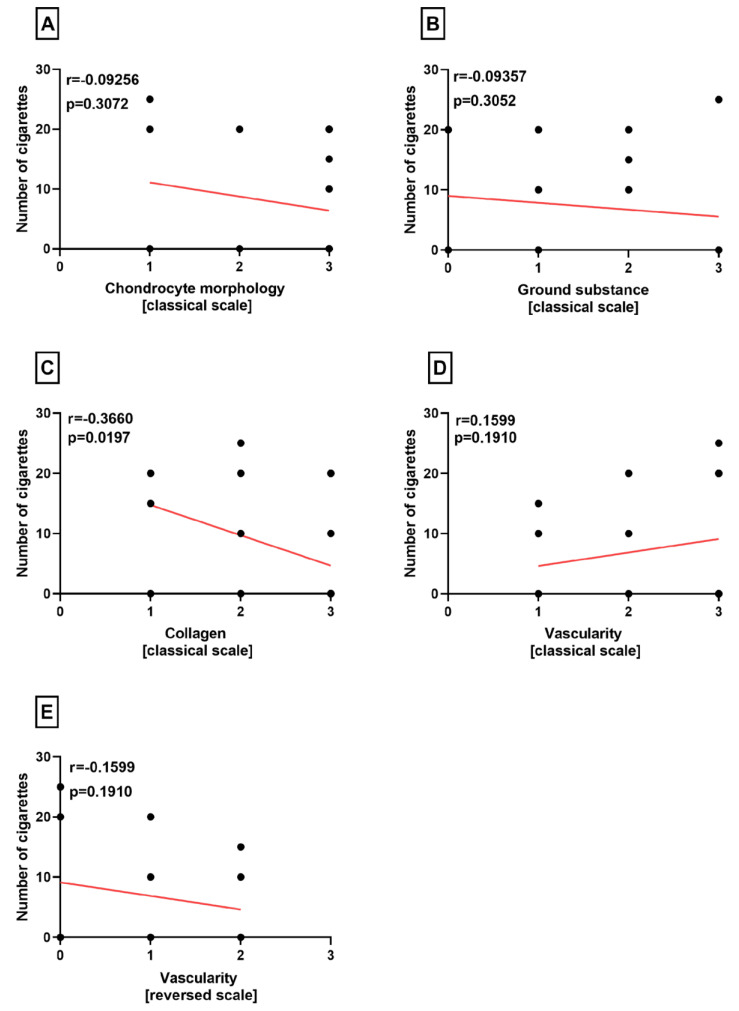
Summarized statistical analysis based on the chondrocyte morphology, ground substance, collagen, vascularity, and reversed vascularity in the **medial menisci**. (**A**) Correlation between the **chondrocyte morphology and number of cigarettes** smoked per day. (**B**) Correlation between the **ground substance and number of cigarettes** smoked per day. (**C**) Correlation between the **collagen composition and number of cigarettes** smoked per day. (**D**) Correlation between the **vascularity and number of cigarettes** smoked per day. (**E**) Correlation between the **reversed vascularity and number of cigarettes** smoked per day.

The three variables of the classical Bonar score (chondrocyte morphology, ground substance, and vascularity) and modified neovascularization variable [14] showed no association with the number of cigarettes in medial menisci (*p* = 0.3072, *p* = 0.3052, *p* = 0.1910, *p* = 0.1910) (Figure 3A,B,D,E). However, there was a statistically significant negative correlation between the number of cigarettes and collagen in the medial menisci (*p* = 0.0197) (Figure 3C).

**Figure 4:**
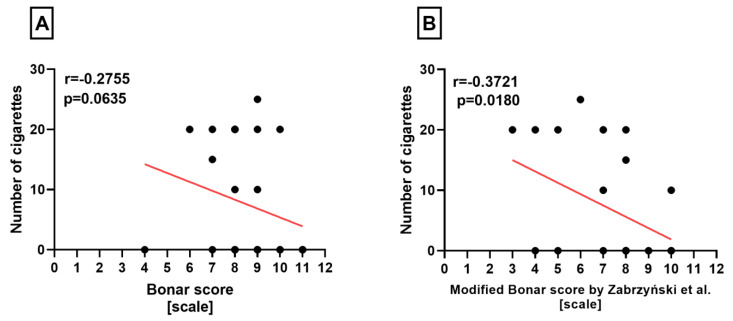
Summarized statistical analysis based on the classical Bonar score and modified Bonar score in the **medial menisci**. (**A**) Correlation between the **classical Bonar score and the number of cigarettes** smoked per day. (**B**) Correlation between the **modified Bonar score [14] and the number of cigarettes** smoked per day.

Additionally, we noted that the modified Bonar score correlated negatively with the number of cigarettes (*p* = 0.0180), but the classical Bonar score showed no association with the daily number of cigarettes smoked, in the medial menisci (*p* = 0.0635) (Figure 4).

**Figure 5:**
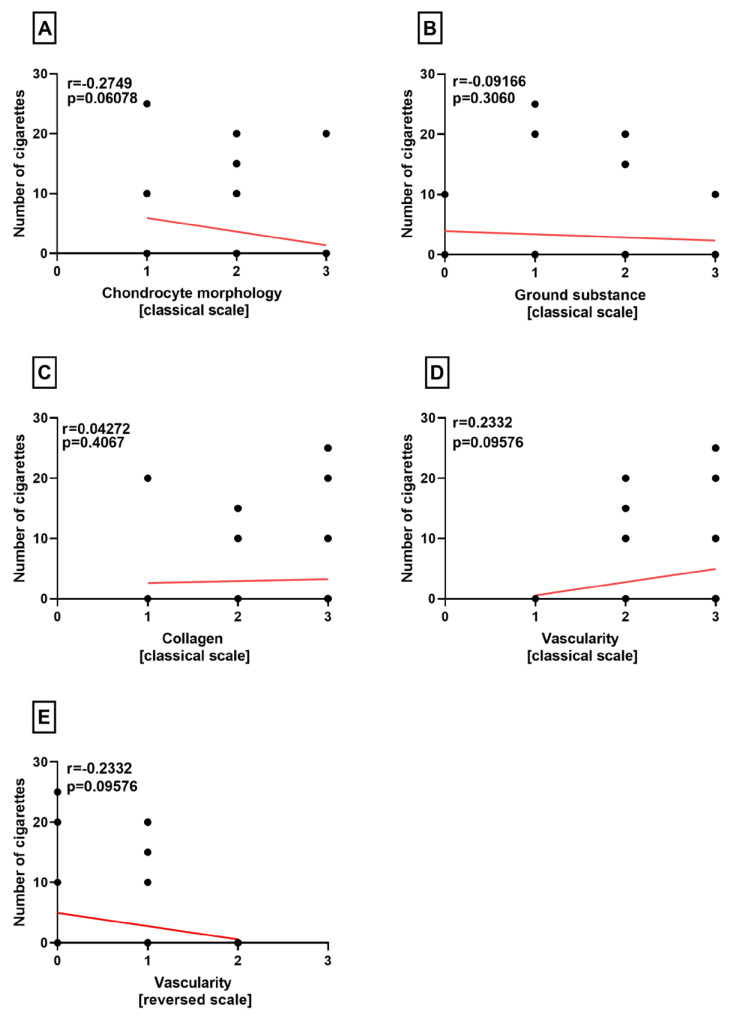
Summarized statistical analysis based on the chondrocyte morphology, ground substance, collagen, vascularity, and reversed vascularity in the **lateral menisci**. (**A**) Correlation between the **chondrocyte morphology and the number of cigarettes** smoked per day. (**B**) Correlation between the **ground substance and the number of cigarettes** smoked per day. (**C**) Correlation between the **collagen composition and the number of cigarettes** smoked per day. (**D**) Correlation between the **vascularity and the number of cigarettes** smoked per day;(**E**) Correlation between the **reversed vascularity and the number of cigarettes** smoked per day.

Furthermore, no correlation was identified between the number of cigarettes and all four variables of the classical Bonar score in the lateral menisci (*p* = 0.06078, *p* = 0.3060, *p* = 0.4067, *p* = 0.09576) (Figure 5A–D). The number of cigarettes did not exhibit a correlation with the modified neovascularization variable [14] as well (*p* = 0.09576) (Figure 5E).

**Figure 6:**
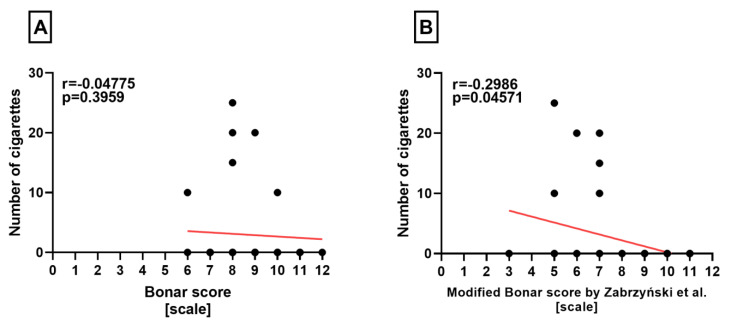
Summarized statistical analysis based on the classical Bonar score and the modified Bonar score in the **lateral menisci**. (**A**) Correlation between the **classical Bonar score and the number of cigarettes** smoked per day. (**B**) Correlation between the **modified Bonar score [14] and the number of cigarettes** smoked per day.

On the other hand, we observed a statistically significant correlation between the number of cigarettes and the modified Bonar score (*p* = 0.04571), but no correlation with the classical Bonar score in the lateral menisci (*p* = 0.3959) (Figure 6A, B).

**Figure 7:**
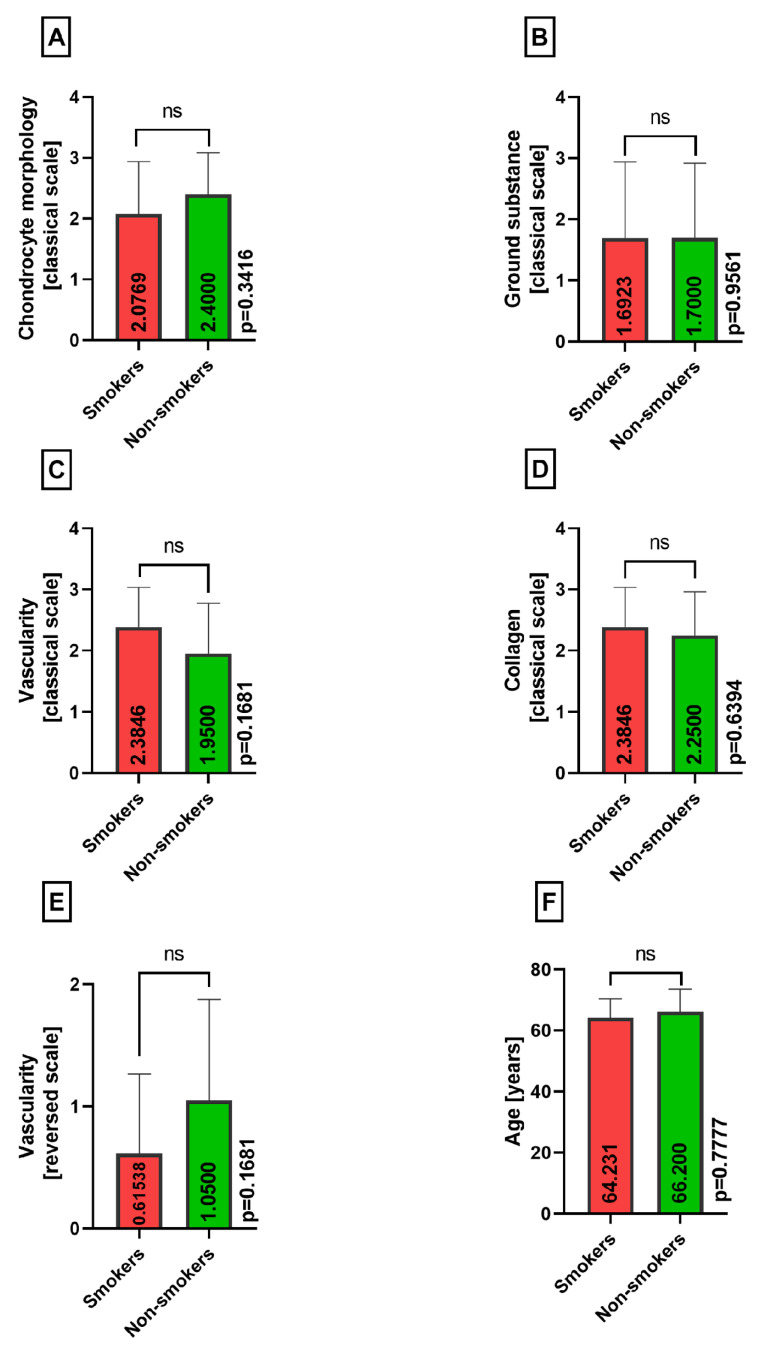
Summarized statistical analysis based on the chondrocyte morphology, ground substance, vascularity, collagen, reversed vascularity, and age in the **lateral menisci** (ns meaning not statistically significant). (**A**) Comparison of the **chondrocyte morphology between the smokers and nonsmokers** groups. (**B**) Comparison of the **ground substance between the smokers and nonsmokers** groups. (**C**) Comparison of the **vascularity between the smokers and nonsmokers** groups. (**D**) Comparison of the **collagen composition between the smokers and nonsmokers** groups. (**E**) Comparison of the reversed **vascularity between the smokers and nonsmokers** groups. (**F**) Comparison of the **age between the smokers and nonsmokers** groups.

Chondrocyte morphology, accumulation of ground substance, vascularity, collagen, age, and modified neovascularization variables did not show any significant difference between the smokers and nonsmokers groups in the lateral menisci (*p* = 0.3416, *p* = 0.9561, *p* = 0.1681, *p* = 0.6394, *p* = 0.7777, *p* = 0.1681) (Figure 7A–F).

**Figure 8:**
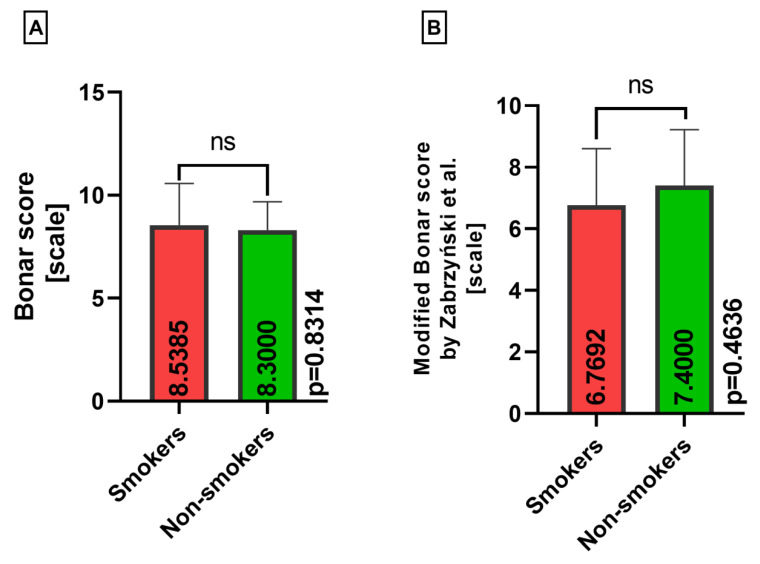
Summarized statistical analysis based on the classical Bonar score and the modified Bonar score in the **lateral menisci** (ns meaning not statistically significant). (**A**) Comparison of the **classical Bonar score between the smokers and nonsmokers** groups. (**B**) Comparison of the **modified Bonar score [14] between the smokers and nonsmokers** groups.

Moreover, there were no statistically significant differences, either in the classical Bonar score or the modified Bonar score, between smokers and nonsmokers for the lateral menisci (*p* = 0.8314, *p* = 0.4636) (Figure 8).

**Figure 9:**
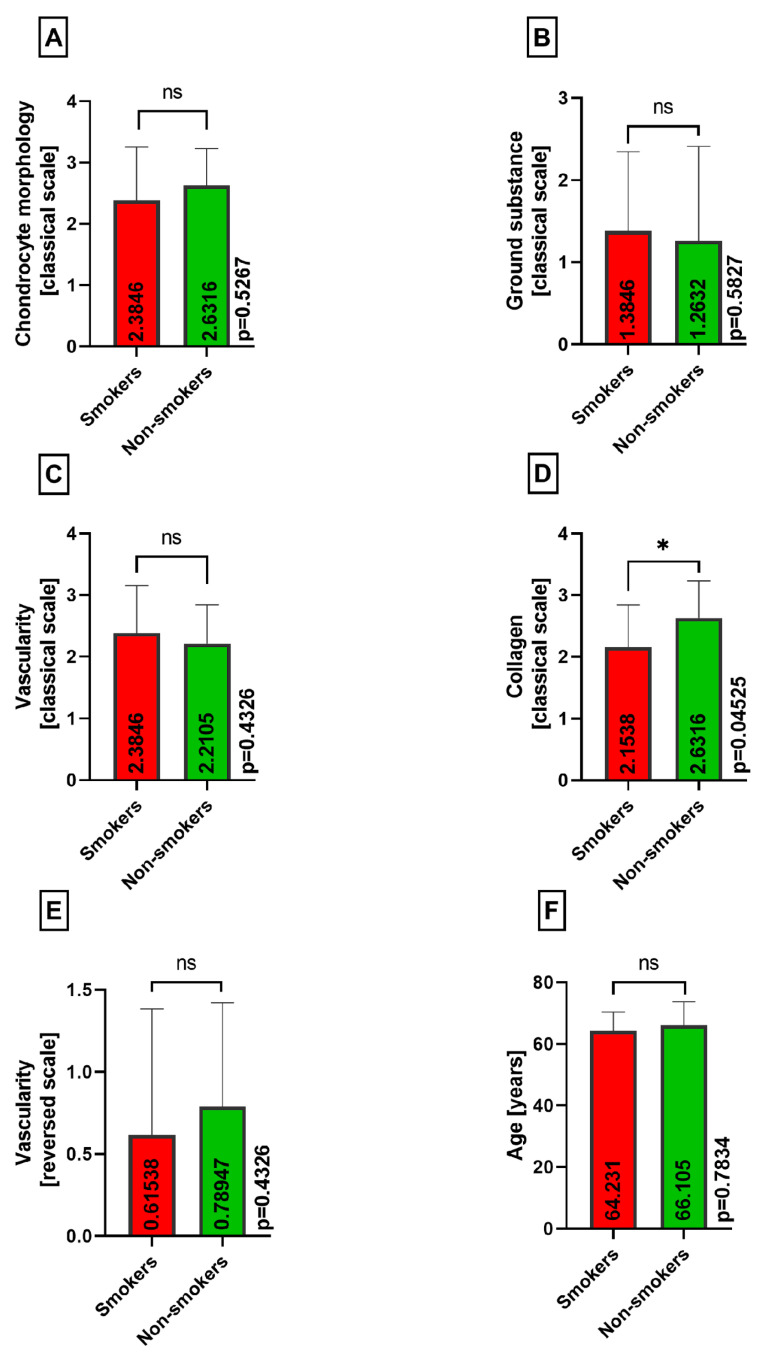
Summarized statistical analysis based on the chondrocyte morphology, ground substance, vascularity, collagen, reversed vascularity, and age in the **medial menisci** (ns meaning not statistically significant and * meaning statistically significant). (**A**) Comparison of the **chondrocyte morphology between the smokers and nonsmokers** groups. (**B**) Comparison of the **ground substance between the smokers and nonsmokers** groups. (**C**) Comparison of the **vascularity between the smokers and nonsmokers** groups. (**D**) Comparison of the **collagen composition between the smokers and nonsmokers** groups. (**E**) Comparison of the reversed **vascularity between the smokers and nonsmokers** groups. (**F**) Comparison of the **age between the smokers and nonsmokers** groups.

Similarly, between smokers and nonsmokers, no statistically significant differences were observed in the chondrocyte morphology (*p* = 0.5267), ground substance (*p* = 0.5827), vascularity (*p* = 0.4326), reversed vascularity (*p* = 0.4326), and age (*p* = 0.7834), assessing medial menisci (Figure 9A–C,E,F). Regarding the collagen variable, there was a statistically significant difference between the smokers and nonsmokers groups, as presented in Figure 9D (*p* = 0.04525).

**Figure 10:**
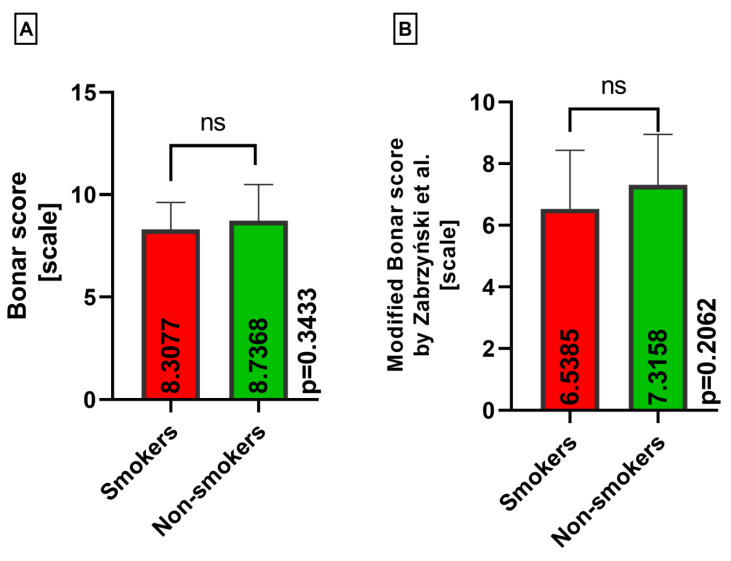
Summarized statistical analysis based on the classical Bonar score and modified Bonar score in the **medial menisci** (ns meaning not statistically significant). (**A**) Comparison of the **classical Bonar score between the smokers and nonsmokers** groups. (**B**) Comparison of the **modified Bonar score [14] between the smokers and nonsmokers** groups.

When comparing the classical Bonar score and modified Bonar score in the medial menisci in terms of smoking status, no statistically significant differences were found (*p* = 0.3433 and *p* = 0.2062) (Figure 10A,B).

**Figure 11:**
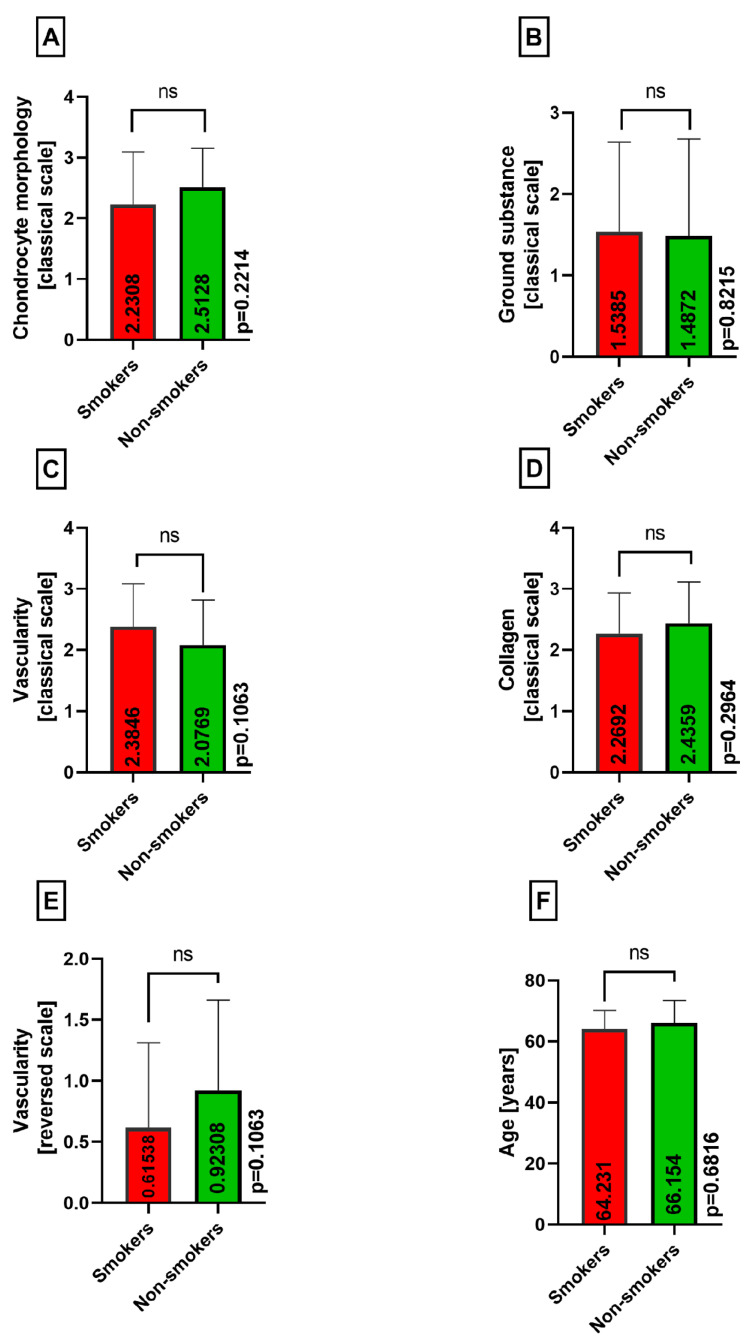
Summarized statistical analysis based on the chondrocyte morphology, ground substance, vascularity, collagen, reversed vascularity, and age **in both menisci** (ns meaning not statistically significant). (**A**) Comparison of the **chondrocyte morphology between the smokers and nonsmokers** groups. (**B**) Comparison of the **ground substance between the smokers and nonsmokers** groups. (**C**) Comparison of the **vascularity between the smokers and nonsmokers** groups. (**D**) Comparison of the **collagen composition between the smokers and nonsmokers** groups. (**E**) Comparison of the **reversed vascularity between the smokers and nonsmokers** groups. (**F**) Comparison of the **age between the smokers and nonsmokers** groups.

Furthermore, there were no statistically significant differences between the smokers and nonsmokers group when comparing the chondrocyte morphology (*p* = 0.2214), ground substance (*p* = 0.8215), vascularity (*p* = 0.1063), collagen (*p* = 0.2964), reversed vascularity (*p* = 0.1063), and age (*p* = 0.6816) in both medial and lateral menisci, as presented in Figure 11A–F.

**Figure 12:**
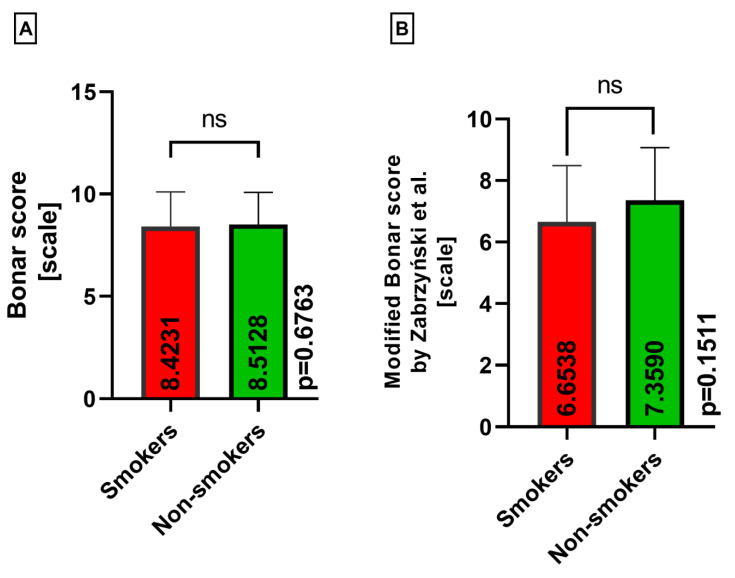
Summarized statistical analysis based on the classical Bonar score and modified Bonar score in **both menisci** (ns meaning not statistically significant). (**A**) Comparison of the **classical Bonar score between the smokers and nonsmokers** groups. (**B**) Comparison of the **modified Bonar score [14] between the smokers and nonsmokers** groups.

Moreover, the classical Bonar score and modified Bonar score variables exhibited no differences based on smoking status in both the lateral and medial menisci (*p* = 0.6763, *p* = 0.1511) (Figure 12A,B).

## 4. Discussion

In our study, we found no evidence of an association between nicotine and the degeneration of meniscal tissue using the Bonar score system. The effect of nicotine on meniscal tissue was quantified using the Bonar scoring system and its modifications in microscopic examination. The most important finding of this study was the negative correlation between the modified Bonar score and the number of cigarettes. To the best of our knowledge, this is the first study aimed at assessing the differences in human meniscal tissue histopathology between smokers and nonsmokers. This study is also another attempt to explore the use of the Bonar score in meniscus pathology.

Nicotine product use has doubtless adverse effects on human health. Numerous studies confirm that tobacco smoking is the main contributor to lung and upper respiratory tract cancer and a significant risk factor for cardiovascular diseases, prostate, and bladder cancers [39,40,41]. It makes tobacco use the leading avoidable cause of death worldwide [39]. The advent of tobacco use associated with e-cigarette vaping seems to postpone the perspective of a nicotine-free society [42,43,44].

The effect of nicotine’s use on the musculoskeletal system is well established, too. It directly toxifies osteoblast and osteoclast activity and indirectly negatively regulates sex and adrenocortical hormones, vitamin D, intestinal calcium absorption, vessels, and oxygen supply [45]. It leads to lower bone mineral density, increased fracture risk, impaired fracture or soft tissue healing, and increased joint disease activity. Moreover, it adversely affects muscles, tendons, cartilage, and ligaments [26,46]. Rose et al. proved that secondhand smoke caused more severe OA in medial meniscus destabilized mice models compared to room-air-exposed mice when assessed by the histopathological Mankin score because of increased expression of proinflammatory molecules [47].

Despite the well-known adverse effects of smoking on soft tissues, few papers have focused on the impact of tobacco on meniscal tissue, providing inconclusive outcomes. Domzalski et al. proved that smokers used to have a lower KOOS score and prolonged time to return to activities of daily living and sport when compared to nonsmokers after outside-in meniscal repair [48]. Blackwell et al. documented the failure rate after meniscal repairs in smokers to be 27%, compared to 7% in nonsmokers [25]. In Uzun et al.’s material, there was a higher failure rate in medial and lateral menisci repairs in smokers than in nonsmokers in the follow-up of 51.2 and 63.2 months, respectively [28,29]. Haklar et al. observed that smoking significantly affected meniscal healing [49]. Overall, many authors highlight the negative effect of smoking on meniscal surgery postoperative outcomes [25,28,29,48,49]. Franovic et al. noted that active smoking not only has an impact on patients’ postoperative outcomes but also their smoking history [50]. On the other hand, Astur et al. found no difference in the quality of life of nicotine users and nonsmokers six months after anterior cruciate or meniscal surgery [51]. Moses et al.’s evaluation proved no association between smoking and failed meniscus repair in bucket-handle tears [32]. Also, Zabrzyński et al. [14] found no connection between smoking indices (smoking years, number of cigarettes per day, pack-year index) and functional outcomes after all-inside repair of chronic medial meniscus tears [23]. Moreover, smoking was not associated with failure after all-inside meniscus repair in Laurendon et al.’s material [31]. What is interesting, on the other hand, is that the literature is quite univocal when it comes to the detrimental effect of cigarette smoking on ligament and tendon health and the results of their reconstructions and repairs [34,52,53,54,55,56,57,58,59].

In this study, we aimed to assess the differences in human meniscal tissue histopathology between smokers and nonsmokers. The comparison did not prove significant differences in the total Bonar score and the new Bonar score in the medial and lateral menisci between both groups. No differences existed in every variable assessed (chondrocyte morphology, ground substance, collagen composition, vascularity score, and reversed vascularity score) in the lateral menisci groups. The only difference in scores was in the collagen organization of medial menisci, which was, surprisingly, more organized in the smoker group. Unexpectedly, the present study showed a weak negative correlation between the number of cigarettes per day and the new Bonar score result. This might suggest that the higher the number of cigarettes smoked daily, the less severe the negative involvement of meniscal histology. It should be pointed out that the correlation is weak, and enrolling more patients in the study could strengthen this correlation.

Park et al. introduced the use of the Bonar score for the first time in assessing meniscus root pathology in knee osteoarthritis. In their study, the mean Bonar score for patients with degeneration was higher (8.5 and 13.5) than those without degeneration (4) [15]. Quite the opposite was true in our study, in which smokers should have a higher Bonar score, although they had an 8.42 and nonsmokers had an 8.51. On the other hand, in our study, the modified Bonar score was lower in smokers than in nonsmokers. This can indicate the much more advanced pathological process of neovascularization, implying that the degeneration process is developed to a lesser extent. However, compared to Park et al.’s results, the more advanced the process of meniscal degeneration, the higher the degree of neovascularization. Moreover, Zabrzyński et al. [14] observed that neovascularization occurs in pathological conditions such as OA in tendons [14]. Noting similarities in the microstructure of fibrous connective tissue between meniscus roots and tendons, it can also confirm that neovascularization is related to a higher degree of degeneration. As mentioned above, our study results showed the opposite.

Nevertheless, this finding, interpreted with caution, might be hypothetically explained by the fact that low-metabolism tissues like menisci may paradoxically benefit from transient ischemia caused by cigarette smoking. Hypoxia caused by tobacco smoke triggers vascular endothelial growth factor (VEGF) synthesis, which is responsible for capillary expansion and stimulates the angiogenesis process in tissues. Many disorders related to smoking: OA, retinopathy, inflammation, tumors, and advanced tendinopathy, are associated with neovascularization. Nicotine plays a major role in the neovascularization process, stimulating endothelial growth, migration, survival, tube formation, and nitric oxide production. This process is described as the pathological formation process of new capillaries with abnormal permeability [38,60,61,62,63], which can be the reason for lower modified Bonar score in smokers group.

Even though pathological, the more significant number of vessels might be attributed to the higher blood flow and partially explain the contraindicatory results in studies that examined the effects of meniscal repair in smokers and nonsmokers. What supports this hypothesis is the relatively low number of cigarettes smoked per day in our groups (the mean of 5.2308), which might be considered a factor contributing to interrupted hypoxia events.

Several limitations were noted in this study. We recommend interpreting our findings cautiously because one of the paper’s limitations is the relatively low menisci in the smokers’ group. If true, these “benefits” are not to overrule the disastrous effect of tobacco smoking on the cardiovascular system and its neoplastic potential. Furthermore, the sample size was modest, with a predominance of female participants and nonsmokers. To create a more homogeneous population, strict exclusion criteria were applied to enhance the statistical power. Additionally, the Bonar score system was developed primarily for tendon pathology instead of meniscus. This could potentially introduce bias in our results.

## 5. Conclusions

The microscopic investigation showed no differences in the menisci of smokers and nonsmokers, except for the collagen, which was more organized in smokers. Moreover, the modified Bonar score was negatively correlated with the number of cigarettes, which supports the role of neovascularization in meniscus pathology under the influence of tobacco smoking.

We want to examine a more extended group, such as 200 patients, as this paper is only the pilot study. Also, the neovascularization issue is very interesting, suggesting that vascularization could be a game changer in meniscus histopathology, as we observed that smokers had an abundant neovascularization process.

## Figures and Tables

**Table 1:** Summary of demographic and clinical characteristics of patients.

Characteristics	Total	Smokers	Nonsmokers
No. of patients	34	13	21
Female	21	7	14
Male	13	6	7
Age	65.385 (54–81)	64.231 (54–72)	66.154 (55–81)
Classical Bonar score	8.4462 (4–12)	8.4231 (6–12)	8.5128 (4–11)
Modified Bonar score	7.0462 (3–11)	6.6538 (3–10)	7.3590 (4–11)

**Table 2:** Summary of meniscus characteristics.

Characteristic	Total	Meniscus	
		Medial	Lateral
No. of samples	65	32	33
No. of cigarettes	5.2308 (0–25)	7.5000 (0–25)	3.0303 (0–25)
Classical Bonar score	8.4462 (4–12)	8.5000(4–11)	8.3939(6–12)
Modified Bonar score	7.0462 (3–11)	6.9375 (3–10)	7.1515 (3–11)

## Data Availability

Data are contained within the article.
